# Sequence Variants and Haplotype Analysis of Cat *ERBB2* Gene: A Survey on Spontaneous Cat Mammary Neoplastic and Non-Neoplastic Lesions

**DOI:** 10.3390/ijms13032783

**Published:** 2012-03-02

**Authors:** Sara Santos, Estela Bastos, Cláudia S. Baptista, Daniela Sá, Christophe Caloustian, Henrique Guedes-Pinto, Fátima Gärtner, Ivo G. Gut, Raquel Chaves

**Affiliations:** 1 Institute for Biotechnology and Bioengineering, Centre of Genomics and Biotechnology, University of Trás-os-Montes and Alto Douro (IBB, CGB-UTAD), Quinta de Prados, 5001-801 Vila Real, Portugal; E-Mails: sarasantos@utad.pt (S.S.); ebastos@utad.pt (E.B.); danielasa@portugalmail.com (D.S.); hgp@utad.pt (H.G.-P.); 2 Department of Genetics and Biotechnology, University of Trás-os-Montes and Alto Douro, Quinta de Prados, 5001-801 Vila Real, Portugal; 3 Department of Veterinary Clinics, Institute of Biomedical Sciences Abel Salazar, University of Porto (ICBAS-UP), Largo Professor Abel Salazar, 2, 4099-003 Porto, Portugal; E-Mail: csbaptista@icbas.up.pt; 4 CEA/DSV/IG-Centre National de Génotypage, Bâtiment G2, 2 rue Gaston Crémieux, CP 5721, 91057 Evry Cedex, France; E-Mails: igut@pcb.ub.es (I.G.G.); caloust@cng.fr (C.C.); 5 Department of Pathology and Molecular Immunology, Institute of Biomedical Sciences Abel Salazar (ICBAS), University of Porto, Largo Prof. Abel Salazar 2, 4099-003 Porto, Portugal; 6 Institute of Pathology and Immunology of the University of Porto (IPATIMUP), Rua Dr. Roberto Frias, s/n, 4200-465 Porto, Portugal; E-Mail: fgartner@ipatimup.pt

**Keywords:** v-erb-b2 erythroblastic leukemia viral oncogene homolog 2 (*ERBB2*), Cat Mammary Tumors (CMT), Sequence Variants (SVs), Haplotypes, Splicing Sites (SS)

## Abstract

The human *ERBB2* proto-oncogene is widely considered a key gene involved in human breast cancer onset and progression. Among spontaneous tumors, mammary tumors are the most frequent cause of cancer death in cats and second most frequent in humans. In fact, naturally occurring tumors in domestic animals, more particularly cat mammary tumors, have been proposed as a good model for human breast cancer, but critical genetic and molecular information is still scarce. The aims of this study include the analysis of the cat *ERBB2* gene partial sequences (between exon 17 and 20) in order to characterize a normal and a mammary lesion heterogeneous populations. Cat genomic DNA was extracted from normal frozen samples (*n* = 16) and from frozen and formalin-fixed paraffin-embedded mammary lesion samples (*n* = 41). We amplified and sequenced two cat *ERBB2* DNA fragments comprising exons 17 to 20. It was possible to identify five sequence variants and six haplotypes in the total population. Two sequence variants and two haplotypes show to be specific for cat mammary tumor samples. Bioinformatics analysis predicts that four of the sequence variants can produce alternative transcripts or activate cryptic splicing sites. Also, a possible association was identified between clinicopathological traits and the variant haplotypes. As far as we know, this is the first attempt to examine *ERBB2* genetic variations in cat mammary genome and its possible association with the onset and progression of cat mammary tumors. The demonstration of a possible association between primary tumor size (one of the two most important prognostic factors) and the number of masses with the cat *ERBB2* variant haplotypes reveal the importance of the analysis of this gene in veterinary medicine.

## 1. Introduction

Naturally occurring tumors in domestic animals that share a similar environment with humans and therefore might be exposed to similar risk factors have been recognized as an interesting opportunity for comparative oncology [1–3]. Cat mammary tumors have been proposed as a good model for human breast cancer (HBC) [1,4–6], mainly because show similar average age at diagnosis, risk factors, histopathology, prognostic aspects (as tumor size and lymph node metastasis) and response to therapy. Recently, Burray and collaborators [7], demonstrated that cat pre-invasive mammary lesions (non-neoplastic and neoplastic), share the full spectrum of morphologic features with pre-invasive mammary lesions in women.

Cat mammary tumors are the third most common neoplasm in domestic cats [8,9]. From cat mammary lesions, approximately 80% are malignant and 10–20% are benign (neoplastic and non-neoplastic lesions) [9]. It is important to be aware that benign lesions can subsequently undergo malignant transformation [9].

Clinical staging of feline tumors is based on the world health organization’s TNM classification for HBC modified to tumors in domestic animals [10]. It is known that tumor size and lymph node metastasis are the two most important prognostic factors, which are also significantly correlated with each other [11]. Additional prognostic information can be gained from histopathology grading, mitotic cell count and by several molecular markers such as *ERBB2* (v-erb-b2 erythroblastic leukemia viral oncogene homolog 2) [6,9,12].

The human *ERBB2* proto-oncogene (also known as HER2, neu) comprises 27 coding exons and encodes a transmembrane tyrosine kinase receptor protein, which is a member of the epidermal growth factor receptor family [13,14]. The HER family share an overall structure that encompass an intracellular carboxy-terminal tail [15,16]. When specific sites in the intracellular domain are phosphorylated, several signaling pathways that contribute to cell division, migration, adhesion, differentiation and apoptosis are activated [16].

*ERBB2* gene amplification and protein overexpression were previously described for HBC [17,18] and cat mammary tumors [6,7,12,19]. Also, *ERBB2* RNA overexpression was demonstrated in 15–25% of HBC cases, and in 55% of cat mammary tumors [6,20–22]. Additionally it is also known that *ERBB2* gene amplification and protein overexpression confer poor prognosis in HBC [17,18]

Genes commonly amplified or deleted often enclose point mutations that activate or inactivate them [23,24]. In recent years, a number of mutational profiling studies have attempted to further identify clinically relevant mutations in HBC. The most notable overall observation is the lack of evidence to support a significant association between *ERBB2* single nucleotide polymorphisms and breast cancer initiation, despite the information supporting its role in breast cancer progression [25–27]. In fact, sequence variants can directly alter the sequence that will be translated into protein, but can also affect splicing and, as a consequence, lead to the appearance of truncated proteins (as previously described for human erbB-2 protein) or to the lack of the correct gene product [28].

Regarding cat mammary lesions, the detection of genomic sequence variants (SVs) was previously reported, but only for *TP53* [29–31] and *TWIST1* genes [32]. Therefore, there is no information concerning the cat *ERBB2* gene sequence variations in this type of lesion.

In the present work, we attempted to analyze the cat *ERBB2* gene SVs in the genomes of cat mammary lesions (which include benign and malignant lesions), determine frequent haplotypes and establish putative associations between SVs and mammary tumor clinicopathological features. Considering this purpose, we partially isolated and sequenced the cat *ERBB2* gene (comprising exons 17 to 20) in normal samples and in mammary lesions. The normal cat genomic DNA was analyzed to detect *de novo* SVs and ascertain the wild type haplotype for comparison with respective genomic DNA sequences from mammary lesions. Moreover, we also performed *in silico* comparative studies with cat and human *ERBB2* DNA, mRNA and protein sequences available in GenBank in order to establish the physical boundaries of cat *ERBB2* gene exons. This analysis allowed the physical localization of SVs in the cat *ERBB2* gene and also the prediction of splicing points.

## 2. Results and Discussion

### 2.1. Extraction of Genomic DNA from FFPET and Frozen Samples

The mammary lesions were clinicopathologically characterized (*n* = 41; Supplementary Table 1), including benign non-neoplastic and neoplastic lesions, primary malignant lesions and metastatic lesions in regional lymph nodes and distant organs. Each mammary lesion was collected from a different cat with the exception of the metastatic samples. (cf. Supplementary Table 1 lesions 40 and 41).

We analyzed 16 control samples (peripheral blood) of which 10 were obtained from cats with mammary lesions and six disease-free cats (Supplementary Table 2). gDNA was obtained from the 16 normal samples and all the 41 mammary lesion samples.

The integrity of the gDNA obtained was confirmed by agarose gel electrophoresis. The gDNA isolated was analyzed in a NanoDrop spectrophotometer and showed satisfactory quality and quantity.

### 2.2. Analysis of Cat ERBB2 Gene DNA Fragments from Exons 17 to 20: Structural Features

Two DNA fragments from cat *ERBB2* gene were amplified in order to analyze the DNA sequence between exons 17 to 20: *ERBB2*_17-18 (343 bp) and *ERBB2*_19-20 (548 bp) fragments.

The PCR products obtained for the 16 normal samples were further sequenced. For each cat *ERBB2* gene sequence fragment (*ERBB2*_17-18 and *ERBB2*_19-20), an alignment was performed with DNA sequences obtained from normal samples. The consensus sequence achieved for each DNA fragment was submitted to GenBank and used as reference: HM132072 (cat-*ERBB2*_17-18; Supplementary Figure 1) and HM196846 (cat-*ERBB2*_19-20; Supplementary Figure 2).

The comparative analysis of cat *ERBB2*_17-18 (GenBank: HM132072) and cat *ERBB2*_19-20 (GenBank: HM196846) DNA sequences, with cat *ERBB2* mRNA (GenBank: AY702651.1), human *ERBB2* mRNA (GenBank: NM_001005862.1) and human *ERBB2* DNA (GenBank: NM_004448.2) sequences, allowed us to define the exon boundaries of the sequence under analysis: exons 17 (cat *ERBB2*_17-18: 1–137 nt), 18 (cat *ERBB2*_17-18: 221–343 nt), 19 (cat *ERBB2*_19-20: 1–90 nt) and 20 (cat *ERBB2*_19-20: 366–548 nt). In a broad analysis, cat *ERBB2* exons 17, 18, 19 and 20 demonstrate respectively 89%, 91%, 93% and 93% similarity with the human counterpart. The cat *ERBB2* intron 17 shows 78% of similarity. Human *ERBB2* intron 19 (715 bp) was revealed to be larger than its counterpart in the cat, with only 275 bp. Nevertheless, a considerable identity was found between homologous sequences, corresponding to cat *ERBB2*_19-20: 91–220 nt (76%) and cat *ERBB2*_19-20: 221–365 nt (64%) (cf. Supplementary Figure 2).

A partial protein sequence was obtained translating the DNA sequence corresponding to *ERBB2* exons 17–20 (cat erbB-2_17-20; cf. Supplementary Figures 1 and 2). The cat-erbB-2_17-20 protein showed 100% similarity with the cat protein from Ensembl (Q49LT4_FELCA). Furthermore, multi-alignment of the cat erbB-2_17-20 with human erbB-2 (NCBI UniProt P04626 partial protein sequence) showed 93.7% identity.

In outline, the exon boundaries assigned in this work for cat *ERBB2* DNA sequences are the same as the ones reported for AY685128.1 DNA sequence and confirmed in the GENSCAN web server.

### 2.3. Sequence Variant Detection in Cat-ERBB2_17-18 DNA Sequences

We were not able to detect any SV in the fragment 17–18 of cat *ERBB2* gene, either in normal (*n* = 16) or mammary lesion (*n* = 41) tissues. Nevertheless, the alignment between our reference sequence for cat-*ERBB2*_17-18 (HM132072) and cat mRNA sequence (AY702651.1) revealed one nucleotide variation at position 74, already identified in the alignments reported above (Supplementary Figure 1). However, this SV detected is a synonymous one.

### 2.4. Sequence Variants Detection in the Cat ERBB2_19-20 DNA Sequences

We were able to analyze a total of 38 sequences for cat *ERBB2* gene 19-20 fragments of which 16 were normal and 22 mammary lesions. In fact, for this fragment, the other 19 mammary lesions could not be sequenced.

The alignment between cat-*ERBB2*_19-20 (GenBank: HM196846) and cat mRNA sequences (GenBank: AY702651.1; exons 19-20) identified the presence of three synonymous SVs at sequences positions 9 nt, 425 nt and 437 nt (Supplementary Figure 2).

**Figure 1:**
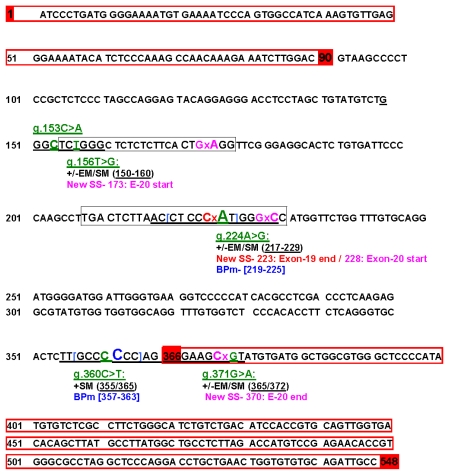
Cat *ERBB2*_19-20 wild type sequence and alternative splicing study. Schematic image regarding the wild type exonic boundaries and alternative splicing sites obtained by Human Splicing Finder (HSF). Red highlighted nucleotide numbers indicate wild type (wt) start and end of exons 19 and 20 (wt exons sequences limited by red boxes). Green/underlying nucleotides (nt) show the five sequence variants detected in the present work. HSF outcome is pointed out by Enhancers Motifs (EM) and Silencers Motifs (SM) splicing sites created (+) or abolished (−). For each SV, the Splicing Sites (SS) and Branch Point motifs (BPm) created or broken are indicated. BP motifs and the respective nucleotide are identifying in blue.

In normal samples (*n* = 16), it was possible to disclose the presence of three genomic SVs: HM196846:g.224A>G, g.360C>T and g.371G>A (Table 1). All three sequence variants were detected in heterozygous condition and the SV g.224A>G was also found in homozygous condition (Table 1). The percentage of allelic variants found in normal samples (Table 1) reinforces the assumption that the DNA reference sequence HM196846 could be considered the genomic wild type (Figure 1 and Supplementary Figure 2).

We analyzed *ERBB2*_19-20 sequences, from 22 cat mammary lesion samples. The multi-alignment of these sequences with our reference sequence (HM196846) allowed the identification of five SVs (Table 1 and Figure 1). Hence, additional to the three SVs detected in the normal samples, two new genomic SVs (HM196846: g.153C>A and g.156T>G) were identified in mammary lesions. It is important to note that, regarding these two SVs, it was not possible to analyze the respective normal samples once most of the material was obtained from the FFPET archive. Therefore, we cannot conclude whether they were somatic mutations or inherited SVs.

From the five genomic variants detected in the cat *ERBB2*_19-20 fragment, four were localized in the intronic region (HM196846: g.153C>A; g.156T>G; g.224A>G and g.360C>T; Figure 1) and one was located at the beginning of exon 20 (HM196846: g.371G>A; Figure 1). Since the allelic change (G to A), corresponds to the modification of codon GCG>GCA which translate to the same amino acid (alanine), the HM196846: g.371G>A is a synonymous sequence variation (detected in normal and cat mammary lesions).

The cat *ERBB2*_19-20 SVs allelic frequencies showed no Hardy–Weinberg equilibrium deviation (Table 1).

Our data, and other from literature demonstrate that there is a great homology between the cat and human *ERBB2* sequences. Taking into consideration this evidence, all sequence variants detected were screened for potential splicing transcripts using Human Splicing Finder (HSF; Figure 1 and Supplementary Table 3).

The *ERBB2*_19-20: g.360C>T SV analysis recognized the wild type splicing site (365/366 nt) with no indication of broken site motif.

Three of the five variants detected showed bioinformatic predictions of aberrant splicing, creating *de novo* splice sites motifs (Supplementary Table 3). The *ERBB2*_19-20: g.156T>G SV create a potential new 3′ splicing site at position 173/174 nt with a possible retention of a large part of intron 19 sequence in exon 20 (Figure 1 and Supplementary Table 3). The variant sequence *ERBB2*_19-20: g.224A>G (position 134 nt of intron 19) creates two new potential splice sites: one at position 223/224 nt with a partial intronic retention generating an alternative end for exon 19, and the other putative new splice site was detected at position 227/228 nt, originating an alternative start for exon 20 with a partial intronic retention (Figure 1 and Supplementary Table 3). Finally, the cat-*ERBB2*_19-20: g.371G>A (position 6 nt of exon 20), showed a new splice site at position 370/371 nt that could create a 3′ upstream exon 20 sequence deletion, originating a small exon 20 (from WT start 366 nt and variant end at 370 nt; Figure 1 and Supplementary Table 3).

Considering the potential branch points, a significant variation between mutant and wild type motifs sequences were detected for two SVs. The g.224A>G SV, localized deeper in intron 19, create a branch point site broken between 219–225 nt. The g.360C>T SV, localized at the end of intron 19, create a putative new site between 357–363 nt that can activate a cryptic splice site, probably in the exon 20 (Figure 1 and Supplementary Table 3). The Human Splicing Finder also enables the analysis of auxiliary splicing sequences as enhancers and silencers. In this case, the sequence variants g.224A>G and g.360C>T, were predicted to create (+) or disrupt (−) a splicing enhancer/silencer by one or more bioinformatic splice prediction tools (Supplementary Table 3). Our results showed an intermediate effect on the splicing efficiency inducing both exon/intron inclusion and skipping (Figure 1; Supplementary Table 3).

### 2.5. Cat ERBB2_19-20 Haplotype Determination in Normal and Mammary Lesion Populations

**Figure 2:**
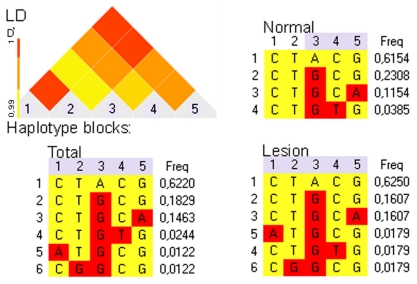
Cat normal and mammary lesion samples haplotypes determination concerning *ERBB2*_DNA 19-20 DNA sequence variants. Haplotype blocks frequency estimated by Linkage Disequilibrium (LD), for normal, lesion and total groups of samples. Triangular graphic corresponds to the total samples LD analysis. Sequence variants are designate in the LD graphic and in haplotype blocks columns: 1-g.[153C>A]; 2-g.[156T>G]; 3-g.[224A>G]; 4-g.[360C>T] and 5-g.[371G>A]. Haplotypes sequences are indicated in blocks lines: 1 = CTACG; 2 = CTGCG; 3 = CTGCA; 4 = CTGTG; 5 = ATGCG; 6 = CGGCG.

The five SVs identified in this work were used for the haplotype determination. We detected four haplotypes in normal samples and six in cat mammary lesions (Figure 2) and noticed that the haplotype showing the higher frequency (h1; Figure 2) corresponds to our reference sequence HM196846. The samples collected from the same animal (normal sample *versus* lesion samples) exhibit the same genetic haplotypes. The most important feature is the identification of specific haplotypes in the cat mammary lesions population, namely haplotypes 5 and 6. As previously reported for the sequence variants analysis, it was not possible to analyze the respective normal samples of the mammary lesions showing the haplotypes 5 and 6.

All variant haplotypes were submitted to GenBank and five accession numbers were provided: HM1968478 (haplotype 2); HM196848 (haplotype 3); HM196849 (haplotype 4); HM196850 (haplotype 5) and HM196851 (haplotype 6).

### 2.6. Statistical Analysis of Cat Mammary Lesions, Clinicopathological Classifiers and Its Association with Haplotypes

All the 41 cat mammary lesions analyzed were clinical and pathologically characterized (Supplementary Table 1 and 2). The Pearsons correlation test between all classifier traits (SPSS software), demonstrated the occurrence of linear correlations between several clinicopathological classifiers (Pearson significant *p*-values ≤ 0.05; Supplementary Table 4).

We observed an outstanding number of correlations that can be interrelated with each other. As example: lymph node invasion *versus* higher vascular infiltration (*p* < 0.01); higher vascular infiltration *versus* higher necrosis presence (*p* = 0.05); higher necrosis presence *versus* higher nuclear/cellular pleomorphism (*p* < 0.01); higher nuclear/cellular pleomorphism *versus* lower number of masses (*p* = 0.02); lower number of masses *versus* no ovary-hysterectomy (*p* = 0.01); no ovary-hysterectomy *versus* less malign lesions (*p* = 0.06); less malign lesions *versus* better clinical outcome (*p* < 0.01) (cf. Supplementary Tables 1 and 4).

In summary, it was possible to highlight important biologically correlations. A strong positive correlation was observed between the mammary lesion pathological grade and the clinical outcome grade. It means that at the same time as the lesion malignity increases, the clinical outcome also worsens (Supplementary Tables 1 and 4). Also, worse clinical outcomes showed a correlation with histological characteristics of malignity, as, for example, a higher grade of vascular infiltration and nuclear/cellular pleomorphism.

In order to test the association between SVs and haplotypes with the mammary tumor clinicopathological features, we allocated the presence or absence of each sequence variant with each classifier trait and for each cat mammary lesion analyzed (cf. Supplementary Tables 1 and 2).

The statistics analysis showed that the g.153T>G SV trend towards an association with the benign neoplastic lesions (χ^2^ *p*-value < 0.05; Fisher exact test *p*-value >0.05 and negative Pearson’s correlation coefficient; Supplementary Table 5).

Taking into account only the primary mammary lesions (benign and malignant), the association test between haplotypes frequency with primary tumor size and with the number of masses (multiple or simple), point out to a putative association that was further scrutinized. For this evaluation we compared the genotypic haplotype frequency for the two classifier traits (Table 2) Considering the primary tumor size (Table 2: T1, T2 and T3), the occurrence frequency of haplotype 1 (*wild type*) is 100% (3/3) for T1; 80% (8/10) for T2 and 50% (5/10) for the T3. The wild type haplotype (haplotype 1) frequency is 53% in neoplastic lesions with multiple number of masses and 83% in neoplastic lesions with few masses.

### 2.7. Discussion

Because erbB-2 protein clearly has an important role in diagnostic and prognosis of breast cancer, the gene encoding it (*ERBB2)* is a natural target for investigation regarding polymorphisms that might indicate resistance or susceptibility for breast cancer development [33,14], reinforced by the idea that polymorphisms might result in increased erbB-2 autophosporylation [25,34,35]. erbB-2 biochemical and genetic studies have revealed that erbB-2 intracellular kinase domain autophosporylation activate the signaling pathways that contribute to cell division, migration, adhesion, differentiation and apoptosis [16,36–38].

In this study, we analyzed two cat *ERBB2* fragments encompassing exon 17 to 20 that encode the following protein regions: outer justamembrane, transmembrane, inner justamembrane and part of the intracellular domain. In these, the intracellular tyrosine kinase region (257 amino-acids), exon 18 encodes for the 3 first amino-acids and exons 19 and 20 are translated into the subsequently 95 amino-acid.

Two reference sequences were accomplished by the analysis of gDNA sequences obtained from normal samples: GeneBank references HM132072 (cat *ERBB2*_17-18) and HM196846 (cat *ERBB2*_19-20).

A comparative analysis between cat and human *ERBB2* DNA sequences from exons 17 to 20 (Supplementary Figures 1 and 2) was also performed. These demonstrated a high percentage of similarity. The exon boundaries assigned in the present work correspond to exons 17–20 from the human *ERBB2*-202 transcript variant that encodes the erbB-2 total size protein. The human and cat erbB-2 proteins also showed a great similarity (93.7%).

Since genome and proteomic browsers are more advanced for humans than for the domestic cat, the DNA, mRNA and protein comparative *in silico* analysis allowed the acquisition of novel knowledge about the cat *ERBB2* gene. Moreover, the great similarity with the human counterpart reinforces the idea that cat mammary tumors could be used as molecular models for HBC.

It is now widely accepted that cancer results from the accumulation of mutations in the genes that directly control cell birth or cell death [39]. In fact, it has been argued that genetic instability is absolutely required for the generation of the multiple mutations that underlie cancer [40]. Single nucleotide polymorphisms represent the most frequent type of genomic sequence variation between individuals. Moreover, their widespread distribution in the genome and their low mutation rate enable the use of single nucleotide polymorphism as genetic markers of diseases phenotypic traits [41].

In the present work, we analyzed one group of cat normal samples (*n* = 16) and another of mammary lesions (*n* = 41). The absence of SVs in the cat ERBB2_17-18 fragment was demonstrated in all normal (*n* = 16) and cat mammary lesion (*n* = 41) gDNA sequences analyzed.

In the cat *ERBB2*_19-20 DNA sequence, it was possible to detect three SVs in the normal and mammary lesion samples: two were localized in intron 19 and one in exon 20 (Figure 1). This last SV (HM196846: g.371G>A) located at the beginning of exon 20 was found to be a synonymous variant. In mammary lesion samples, it was possible to detected two additional SVs (HM196846:g.153C>A and g.156T>G) located in intron 19 (Table 1 and Supplementary Figure 2).

It is known that splicing changes can lead to protein splice isoforms, namely the appearance of truncated proteins (already shown for the human erbB-2 protein) that have an active role in mammary tumors [42]. Since the *ERBB2* gene seems to be conserved in the cat and human genomes, we *in silico* predict the effect of each sequence variant in the splicing, using the bioinformatic tool Human Splice Finder (HSF) [28]. From the five SVs detected in the present work, four were found to be good candidates for the occurrence of alternative splicing: HM196846: g. 156T>G; g.224A>G; g. 360C>T and g.371G>A (Figure 1 and Supplementary Table 3). The *in silico* data obtained was found to be very promising, enhancing the value of the SVs detected. This, however must be experimentally confirmed future studies.

The SVs detected were also analyzed for haplotype determination in order to genetically evaluate the population under study. The cat mammary lesion population showed more genomic sequence variations (64%; five SVs) than the normal one (56%; three SVs). The five SVs pairwise linkage disequilibrium analysis allowed the identification of six haplotypes in the total population, of which four haplotypes were observed in the normal population (Figure 2). The most frequent haplotype 1 (Figure 2), that corresponds to cat-*ERBB2*_19-20 reference sequence (Genebank: HM196846), can be considered the *major ancestral haplotype*. In outline, the genetic analysis of the population in study (*i.e.*, percentage of genomic variation and haplotype frequency) revealed that the cat mammary lesion population presents more genomic instability than the normal one.

Our data unveil several linear correlations between clinicopathological traits. A strong positive correlation was observed between the mammary lesion pathological grade and the clinical outcome grade. The cases with no ovary-hysterectomy presence showed a strong correlation with hormonal treatment presence, and also with higher primary lesions mass number, lower detection of necrosis and lower grade of nuclear/cellular pleomorphism. All of these clinicopathological correlations are known to be in concordance with occurrence of less malignant lesions. Also in agreement were the Pearson correlation results, which also detected a nearly-significant relationship between cases with no ovary-hysterectomy presence and lower levels of malignity (hyperplasic and neoplastic benign lesions). In fact, fibroadenomatous hyperplasia is usually seen in young, sexually intact queens at the time of puberty and also in cats receiving prolonged hormonal therapy (megestrol acetate or medroxyprogesterone acetate) used as contraceptives in females [43], and characterized by aggressive, rapid and abnormal proliferation of one or more mammary glands with progression to necrosis if left untreated [44,45]. In addition, fibroadenoma can evolve from one to all of the mammary glands [46]. Our data is therefore in agreement with the one reported in the literature. As far as we know, it is the first time that such extensive evaluations between clinicopathological traits have been undertaken in cat mammary lesions.

In order to recognize a potential association between the SVs and haplotypes, some clinicopathologic staging traits of all the 41 cat mammary lesions analyzed in the present work were clinically and pathologically characterized (Supplementary Table 1). The HM196846:153T>G SV, only detected in cat mammary lesions, showed a moderate association with the benign neoplastic lesions.

The multiple mammary lesion masses in cats are quite frequent [5]. They are often considered to be associated with hematogenous or lymphogenous involvement [3]. Besides, it is known that tumor size is one of the most important prognostic factors, the larger size being associated with an advanced stage of pathology [11,47,48]. Regarding only the primary lesion samples, our results demonstrated a presumed association between the genotypes with haplotypes that are not wild type (haplotypes 2, 3, 4, 5, 6) with multiple numbers of masses and higher primary tumor size (Table 2). Future studies are mandatory to confirm these putative associations between sequence variants with malignant tumors and between haplotypes with primary tumor size and with multiple numbers of masses.

## 3. Experimental Section

### 3.1. Biological Material

In this study we used 22 formalin-fixed paraffin-embedded tissues (FFPET) and 19 frozen samples, corresponding to a total of 41 mammary lesions obtained from 40 different cats (with an exception for metastasis samples; Supplementary Table 1). 16 blood samples (normal samples), also analyzed, were recovered during mastectomy treatment (*n* = 10) or other routine clinical procedures (*n* = 6) and immediately frozen to preserve the DNA (Supplementary Table 2). Accordingly, ten normal and mammary lesions samples were collected from the same individuals.

The frozen samples were kindly provided by “Trás-os-Montes Veterinary Hospital” and the “UPVet Small Animal Clinics of the Institute of Biomedical Sciences Abel Salazar, University of Porto (ICBAS-UP)”. All FFPET samples belong to a collection of the Veterinary Pathology Laboratory of ICBAS-UP and to the Institute of Molecular Pathology and Immunology of the University of Porto (IPATIMUP).

All tissues were evaluated by pathological procedures. Cat mammary lesion biopsies correspond to a heterogeneous group of samples (non-neoplastic, malignant and benign neoplastic lesions). The histological classification was undertaken in accordance with the Misdorp and collaborators (1999) classification for cat mammary tumors [49].

All experimental work was conducted according to ethical procedures approved (EC/12-04/POCI/CVT/62940/2004; approval date from 02/12/2004 and beginning of the study in 01/07/2005) and was carried out in accordance with best practice and the laws of the countries involved.

### 3.2. Genomic DNA Extraction

The genomic DNA (gDNA) extraction from the FFPET was performed according to the protocol previously described by Santos and collaborators [50]. The isolation of gDNA from normal blood samples was performed with the QuickGene DNA Blood Kit S (Fujifilm Life Science) following the procedures described by the manufacturer.

The gDNA extracted from all samples was analyzed by agarose gel electrophoresis (1% agarose gel, stained with ethidium bromide) in order to evaluate its integrity. Afterwards, the quantity and quality were measured in a NanoDrop ND-1000 spectrophotometer (NanoDrop Technologies).

### 3.3. PCR Amplification of Two Fragments from Cat ERBB2 Gene Comprising Exons 17 to 20

This study focuses on cat *ERBB2* DNA sequence from exons 17 to 20 that encodes for a part of the outer justamembrane region (exon 17), the transmembrane region (exon 17), the inner justamembrane region (exon 17 and 18) and the intracellular region including part of the kinase domain (exons 18, 19 and 20). The four primers used were designed, based on two cat DNA sequences from the National Center for Biotechnology Information (NCBI) genome browser: *ERBB2* mRNA (GenBank: AY702651.1) and *ERBB2* gene partial sequence (GenBank: AY685128). The primer sequences were as follows: sense 5′-CTGTGACGTCCATCATTGC-3′(E17), antisense 5′-CTTGTAGACAGTGCCA AAAGC-3′(E18), sense 5′-ATCCCTGATGGGGAAAATGT-3′(E19) and antisense 5′-GGCAATCTG CACACACCA-3′(E20). Two DNA fragments were amplified and sequenced with two sets of primers, covering the exons 17–18 (primer set E17-E18; 343 bp sequence) and exons 19–20 (primer set E19-E20; 548 bp sequence).

The PCR amplifications made using gDNA from frozen and FFPET samples were performed in a termocycler (T-personal, Biometra) with standard reaction mixtures. The cycling conditions used were: initial denaturation of 5 min at 94 °C, followed by 30 cycles (denaturation for 1 min at 94 °C, annealing for 45 s at 50 °C and extension for 1 min at 72 °C) and one final extension step of 10 min at 72 °C. The PCR products were analyzed by electrophoresis in 1.5% agarose gel, stained with ethidium bromide and visualized under ultraviolet light.

### 3.4. Sequencing and Analysis of DNA Fragments

The fragments obtained were further sequenced in both directions, applying the same set of primers used during PCR amplification. The sequences were analyzed in Vector NTI 10.3.0 software (Invitrogen Life Technologies) using the ContigExpress and Align modules.

A search of the two final DNA reference sequences was performed using the bioinformatic resource BLAST (Basic Local Alignment Search Tool) of NCBI and Ensembl genome browsers. The predicted protein sequence was done with the NCBI bioinformatic resource BLASTP 2.2.21. The search for *ERBB2* exon structure was performed in NCBI and Ensembl genome browsers and in GeneCards database (version 3).

Sequence variants identification nomenclature is in accordance with the standard nomenclature recommendations of the Human Genome Variation Society [51–53].

### 3.5. Bioinformatic Splice Prediction Based on the SVs Detected

The database Human Splicing Finder (version 2.4) was used to predict the effect of each sequence variant on mRNA splicing sites (splicing donor/acceptor sites and branch point sequences) and to accurately predict disruption of splicing enhancer or silencer sites [28]. The consensus values (CVs) for wild type and variant affected splice sites and splice regulatory sites and the variations of the consensus values (ΔCV) obtained for the wild type and the variant motifs were analyzed. It is important to notice that the sequence variants that showed predictions with CVs higher than 70–80 and/or ΔCV reductions of at least 10%, are likely to affect splicing [28]. As a result, only SVs that demonstrated these values were considered for this analysis. With respect to branch point (BP) sequences, the HSF software only processes a 100 bp window and excludes branch point positions −12 nt to −1 nt from the exon beginning.

### 3.6. Statistic Analysis in Normal and Mammary Lesion Cat Populations. Testing Through DNA Variant Sequences and Clinicopathological Features

For genomic SVs detected by experimental analysis, the allele frequency deviations from Hardy–Weinberg equilibrium (HWE) were evaluated by Chi-square test (significant *p*-value < 0.05; SNPAlyse software; DYNACOM).

Pairwise linkage disequilibrium was estimated by a D' absolute value for all sequence variants. Based in linkage disequilibrium and Chi-square tests, haplotype frequencies were estimated for each group (normal and mammary lesions) and for the total population. This analysis was performed in the SNP and Variation Suite™ 7 (Golden Helix) and Haploview 4.2 software applications.

The mammary lesions were clinicopathologically characterized with several tumor classifiers, such as histological grading, clinical staging, other pathological features and clinical outcome. For each classifier score linear type traits were established (higher score values were set for higher grade of malignity). Pearson correlation test allows the identification of significant linear correlations and also provide the orientation of that relation. Correlation analysis was completed between the different classifier traits in the SPSS version 17.0 (significant *p* values > 0.05).

In order to disclose putative associations between SVs and haplotypes with mammary tumor clinicopathological features, we allocated the presence or absence of each sequence variant and haplotype with each classifier trait and for each cat mammary lesion analyzed (cf. Supplementary Tables 1 and 2).

A Pearson correlation test and Crosstabs two-way and multi-way tables were performed. The Crosstabs two-way tables provide the chi-square test (Pearson’s χ^2^ Tests) and Fisher’s exact test as measures of association (SPSS version 17.0 software). The chi-square test was used to test associations of the allelic and haplotype frequency between groups or subgroups of samples (χ^2^ Tests significant *p*-value < 0.05). The Fisher’s exact test correct the association test in the cases were at least one expected cell count is less than 5 (significant *p*-value < 0.05). We also applied Pearson linear correlation test.

## 4. Conclusions

In human breast cancer, several studies considering the analysis of *ERBB2* gene sequence variants were performed focusing on specific polymorphisms already recognized to be possibly associated with HBC pathology [27,54]. On the other hand, cat *ERBB2* gene molecular data is very scarce, including limited information concerning specific SVs, namely in mammary lesions. The present study encloses the sequencing of large DNA fragments in order to identify *de novo* SVs that could be involved in cat mammary tumor onset and progression. Our work focuses in two cat *ERBB2* genomic fragments, embracing exons 17 to 20. We detected sequence variants in the genomic fragment between exon 19 to 20 that encode for 95 amino-acids of the kinase domain. Our results showed a higher rate of SVs in the genomic fragments that encode for this protein region responsible for its accurate activation. In addition, the higher frequency of SVs in mammary lesions compared to the normal samples suggests that the occurrence of SVs in the *ERBB2*_19-20 fragment have a possible impact in the function of the erbB-2 protein. The detailed description of the experimental and *in silico* analysis performed intend to be helpful in future studies on this matter.

The bioinformatics analysis with the Human Splicing Finder database predicted that four of the SVs can produce alternative transcripts that justify further investigation at the experimental level.

Our data demonstrated the occurrence of linear correlations between several clinicopathological traits and also showed a putative association between the five non-wild type haplotypes and clinicopathological traits, namely with primary tumor size and number of masses.

It is important to highlight that, as far as we know, the present study is the first report concerning cat *ERBB2* gene analysis in normal samples and mammary lesions with special emphasis to the detection of sequence variants, alternative splicing sites prediction, haplotypes disclosing and SVs association with clinicopathological traits.

## Supplementary Information



## Figures and Tables

**Table 1:** Sequence variants detected in cat *ERBB2*_19-20 fragment in normal and mammary lesion samples: Hardy-Weinberg equilibrium test. Genomic SVs presence (+) or absence (0). (wt) wild type allele. (*n*) number of samples with the same genomic sequence. (%) Allelic frequency. Hardy-Weinberg equilibrium significant *p*-value < 0.05.

A: cat-*ERBB2*_19-20 genomic sequence variants presence (+) or absence (0)														
Normal DNA sequences (*n* = 16):														
SVs			*n* = 7		*n* = 1		*n* = 3		*n* = 2		*n* = 1		*n* = 2	
g.[224A>G] + [wt]			0		0		**+**		0		**+**		**+**	
g.[224A>G] + [224A>G]			0		**+**		0		**+**		0		0	

g.[360C>T] + [wt]			0		0		0		0		**+**		0	

g.[371G>A] + [wt]			0		0		0		**+**		0		**+**	

**Mammary Lesions DNA sequences (***n***= 22):**														
**SVs**			*n***= 8**		*n***= 5**	*n***= 1**		*n***= 1**	*n***= 1**		*n***= 4**	*n***= 1**		*n***= 1**

g.[153C>A] + [wt]			0		0	0		0	0		0	0		**+**

g.[156T>G] + [wt]			0		0	**+**		0	0		0	0		0

g.[224A>G] + [wt]			0		**+**	**+**		0	**+**		**+**			**+**
g.[224A>G] + [224A>G]								**+**				**+**		

g.[360C>T] + [wt]			0		0	0		0	**+**		0	0		0

g.[371G>A] + [wt]			0		0	0		**+**	0		**+**	0		0

**B: cat-***ERBB2***_19-20 genomic sequence variants frequency and Hardy-Weinberg equilibrium test**														
**Allele frequencies (%):**														
allele	**g.[153C>A]**		allele	**g.[156T>G]**		allele	**g.[224A>G]**		allele	**g.[360C>T]**		allele	**g.[371G>A]**	
				
	Lesion	Normal		Lesion	Normal		Lesion	Normal		Lesion	Normal		Lesion	Normal
									
C	97.7%	100%	T	97.7%	100%	A	36.4%	62.5%	C	97.7%	96.9%	G	88.6%	87.5%
A	2.3%	0%	G	2.3%	0%	G	63.6%	37.5%	T	2.3%	3.1%	A	11.3%	12.5%

**Hardy-Weinberg equilibrium:**														

*p***-value**	**g.[153C>A]**		*p***-value**	**g.[156T>G]**		*p***-value**	**g.[224A>G]**		*p***-value**	**g.[360C>T]**		*p***-value**	**g.[371G>A]**	
				
	Lesion	Normal		Lesion	Normal		Lesion	Normal		Lesion	Normal		Lesion	Normal
				
	0.12E−6			0.12E−6			0.7	0.5		0.12E−6	0.31E−4		0.75	0.37

**Table 2:** Cat mammary primary lesion genotype and their relationship with size and the number of masses. Each diploid genotype is designated by its constituent haplotypes.

Number of Masses	Primary Tumor Size				Diploid Genotype

	T?	T1	T2	T3	
Multiple	1 MaL *		2 MaL		h1/h1
	1 BeL *				h2/h2
			1 MaL		h1/h6
	1 BeL #				h1/h5
				1 MaL	h1/h4
	2 MaL *				h1/h3
		3 MaL	1 MaL	1 BeL	h1/h2
				1 MaL	h2/h3

Simple			1 MaL	1 MaL	h1/h1
				1 MaL	h1/h2

Haplotypes: (h1) CTACG; (h2) CTGCG; (h3) CTGCA; (h4) CTGTG; (h5) ATGCG; (h6) CGGCG. Primary tumor size: (T1) < 2 cm diameter; (T2) 2–3 cm diameter; (T3) > 3 cm diameter; (T?) unknown size. (MaL) malignant lesions; (BeL) benign lesions;

* Frozen samples;

# FFPET samples.

Primary tumor size classification is in agreement with the 1980 World Health Organization’s TNM system, modified to tumors in domestic animals [10].
