# Supplementary material for: Sequence Variants and Haplotype Analysis of Cat ERBB2 Gene: A Survey on Spontaneous Cat Mammary Neoplastic and Non-Neoplastic Lesions
Source: Int J Mol Sci. 2012 Mar 2;13(3):2783–800. doi: 10.3390/ijms13032783 (PMC3317687; doi:10.3390/ijms13032783)
Supplement: Supplementary file 1 [file ijms-13-02783-s001.doc]

Supplementary Information

**Supplementary Figure 1.** Alignment from exon 17 to 18 of the cat and human *ERBB2* DNA and mRNA sequences. The protein sequence obtained by translation of
cat-*ERBB2*_DNA exons 17 and 18 sequences. The cat *ERBB2* mRNA (GenBank: AY702651.1), cat *ERBB2* DNA (cat *ERBB2* partial DNA sequence; GenBank: HM132072), human *ERBB2* mRNA (GenBank: NM_001005862.1) and human *ERBB2* DNA gene (GenBank: NM_004448.2), from exon 17 to 18, aligned in Vector NTI 10.3.0 software (Invitrogen Life Technologies). Black boxes indicate Primer E17 and E18 recognition sequences. Green arrowheads point to sequence variants detected between reference sequence (GenBank: HM132072) and cat *ERBB2* mRNA (GenBank: AY702651.1).


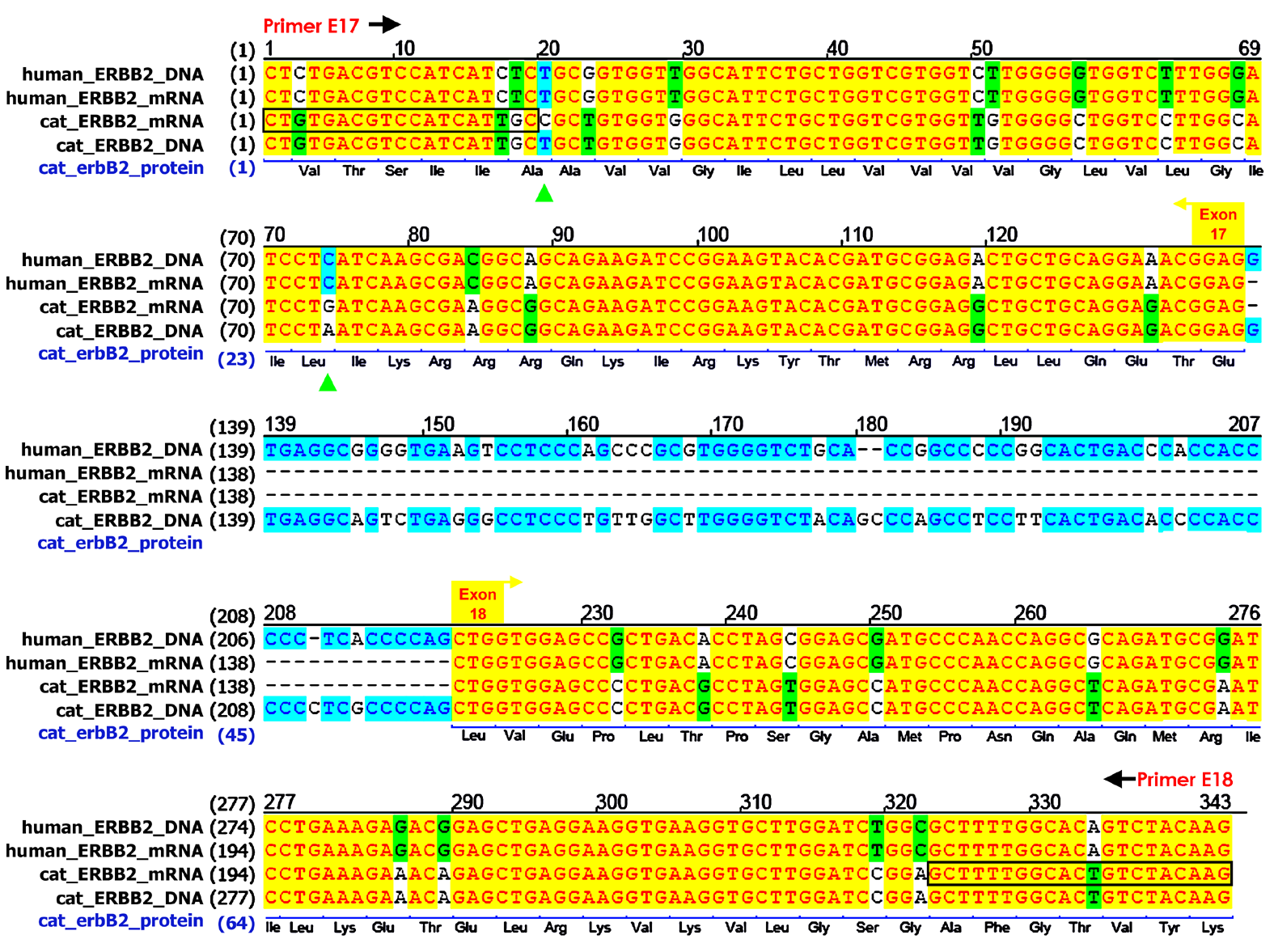


**Supplementary Figure 2.** Alignment of cat and human *ERBB2* DNA and mRNA sequences from exons 19 to 20. Composite image of alignments between six *ERBB2* partial sequences: *ERBB2* mRNA exons 19 and 20 sequences of cat (GenBank: AY702651.1) and human (GenBank: NM_001005862.1); *ERBB2* DNA sequence from cat (GenBank: Gene1918) and human (GenBank: NM_004448.2) Red boxes indicate primer E19 and E20 recognition sequences. Green arrowheads indicate sequence variants detected between cat DNA and mRNA sequence. The protein sequence in blue was obtained by translation of cat-*ERBB2*_DNA exons 19 and 20 sequences.


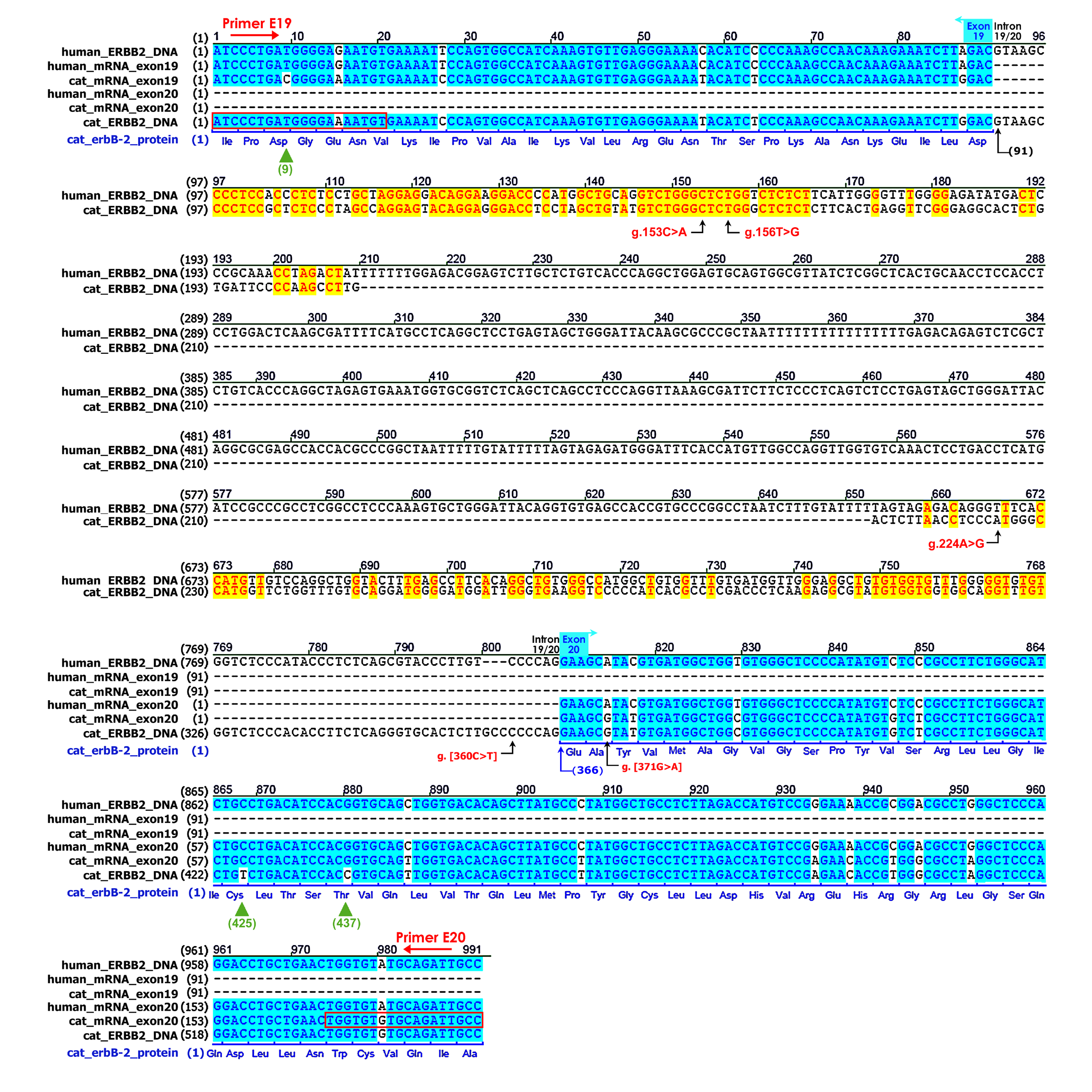


**Supplementary Table 1.** Cat mammary lesions samples clinicopathological evaluation. The 41 cat mammary lesions samples are group by lesion type: Benign; Primary Malignant and Metastasis. The histological classification is according to Misdorp and collaborators (1999) classification for cat mammary tumors [49]. Primary tumor size classification (T1/T2/T3) and lymph node invasion (N0/N1)is in agreement with the 1980 World health organization’s TNM system, modified to tumors in domestic animals [10]. (NE) lymph node non-evaluate. Some clinicopathological evaluation is specified, including the clinical outcome. (*) Cases with blood DNA samples also analyzed. (#) Different lesions samples from the same case but different tissues. (Hyp) Hyperplasia; (Carci.) Carcinoma; (D.S.) Domestic shorthaired; (DC) Developed Carcinoma; (NA) non available data; (DOD) Death by disease, (NED) No evidence of disease, (DOC) Death by other causes.

| **Cases DNA Samples**  **(n = 41)** | **Histological classification** | **Age**  **(years-old)** | **Race** | **Ovary-hysterectomy** | **Hormonal treatment** | **Lesion size grade [49]** | **Number mitosis / field** | **Nuclear / Cellular pleomorphism** | **Number of masses** | **Lymph node invasion** | **Vascular infiltration** | **Necrosis** | **Clinical Outcome** |
| --- | --- | --- | --- | --- | --- | --- | --- | --- | --- | --- | --- | --- | --- |
|  | **Benign Lesions** | | | |  |  |  |  |  |  |  |  |  |
|  | **Non-neoplastic Benign Lesions** | | | |  |  |  |  |  |  |  |  |  |
| 1 * | Fibroadenomatous Hyp. | 6 | D. S. | No | Yes | T1 | 1 | No evidence | Multiple | NE |  | Negative | DC |
| 2 * | Epithelial Hyperplasia | 13 | D. S. | No |  | T1 |  | No evidence | Multiple | NE | Negative | Negative |  |
| 3 | Fibroadenomatous Hyp. | 5 | D. S. |  | Yes | T1 |  | Low |  |  |  |  |  |
| 4 | Fibroadenomatous Hyp. | 6 | Siamese | No | Yes |  |  | Low |  |  |  |  |  |
| 5 | Fibroadenomatous Hyp. | 2.5 | D. S. | No | No | T3 | 1 | No evidence | Multiple |  |  |  |  |
|  | **Neoplastic Benign Lesions** | | | |  |  |  |  |  |  |  |  |  |
| 6 * | Fibroadenomatous Hyp. | 10 | D. S. |  |  | T3 |  | No evidence | Multiple | NE |  |  | NED |
| 7 * | Fibroadenoma |  |  |  |  |  |  |  | Multiple | NE |  |  | DOC |
| 8 | Low-cellularity Fibroadenoma | 9 | D. S. |  |  |  |  |  | Multiple | N0 |  |  |  |
|  | **Primary Malignant Lesions** | | | |  |  |  |  |  |  |  |  |  |
| 9 | Carcinoma | 18 |  |  |  |  |  |  | Multiple |  |  |  |  |
| 10 | Carcinoma | 15 |  |  |  |  |  |  | Multiple |  |  |  |  |
| 11 * | Carcinoma |  |  |  |  |  |  |  | Multiple |  |  |  |  |

***Supplementary Table 1.*** *Cont.*

| 12 | Carcinoma |  |  |  |  |  |  |  |  | N1 |  |  |  |
| --- | --- | --- | --- | --- | --- | --- | --- | --- | --- | --- | --- | --- | --- |
| 13 | Papillary carcinoma | 15 |  |  |  | T2 |  |  | Multiple | N1 | Positive |  |  |
| 14 | Papillary carcinoma | 6 | D. S. |  |  | T2 |  | Moderate-Marked | Multiple |  |  |  |  |
| 15 * | Tubular carcinoma | 8 | D. S. | Yes | Yes | T2 | 1 - 2 | Marked | Single | N0 | Negative | Positive | NED |
| 16 * | Tubulopapillary Carci. |  | Siamese | Yes | No | T2 |  |  | Multiple | NA | Positive | Positive | DOD |
| 17 * | Tubulopapillary Carci. | 14 | D. S. | No | Yes | T1 |  | Low | Multiple | NA | Negative | Negative | DOC |
| 18 | Tubulopapillary Carci. | 13 | D. S. | Yes |  | T1 | 3 - 4 |  |  |  |  | Positive |  |
| 19 | Tubulopapillary Carci. | 7 | D. S. | Yes | No | T2 | 2 - 3 |  | Multiple |  |  | Positive | DOD |
| 20 | Tubulopapillary Carci. | 12 | Siamese | Yes | No | T3 | 8–14 | Marked | Single | N0 |  |  |  |
| 21 | Tubulopapillary Carci. | 8 | D. S. |  |  | T1 | 7 | Marked | Multiple |  |  |  |  |
| 22 | Tubulopapillary Carci. | 6 | D. S. | No | Yes | T1 | 1 - 2 | Low | Multiple | N0 | Negative | Negative |  |
| 23 * | Tubulopapillary/solid Carci | 10 | D. S. | Yes | No | T2 |  | Marked | Single | N1 | Positive | Positive | DOD |
| 24 | Tubulopapillary/solid Carci | 15 | D. S. |  |  | T3 | 8 | Moderate | Multiple | N0 |  | Positive | DOD |
| 25 | Tubular/solid Carci. | 10 |  | No | Yes | T3 | 4 | Low-Moderate | Multiple | N1 | Positive | Positive | DOD |
| 26 | Solid carcinoma | 12 | Siamese |  |  | T2 |  | Moderate |  |  |  |  |  |
| 27 | Solid carcinoma |  | Siamese | No | Yes |  | 2 - 4 | Marked | Single |  |  |  |  |
| 28 | Solid carcinoma | 10 | D. S. | No | Yes | T3 | 5 | Marked | Single | N1 |  | Positive |  |
| 29 | Solid carcinoma | 16 |  |  |  |  | 5 | Marked | Multiple | N1 |  | Positive |  |
| 30 | Solid carcinoma | 9 | D. S. | No | No | T2 | 5 | Moderate | Multiple | N0 | Negative | Positive |  |
| 31 | Solid carcinoma | 12 | D. S. | Yes |  | T2 | 14 | Marked | Multiple |  | Positive |  |  |
| 32 | Solid carcinoma | 13 | D. S. | No |  | T3 | 8 | Moderate | Multiple |  | Positive |  |  |
| 33 | Solid carcinoma | 12 |  | Yes | No | T2 | 4 - 6 | Marked | Single | NA | Positive | Positive |  |

**Supplementary Table 1.** *Cont.*

| 34 | Solid carcinoma | 7.5 | D. S. | Yes |  | T1 | 7 | Low-Moderate | Single |  |  | Positive |  |
| --- | --- | --- | --- | --- | --- | --- | --- | --- | --- | --- | --- | --- | --- |
| 35 | Solid carcinoma |  |  |  |  | T3 | 7 | Moderate-Marked | Multiple | N0 |  |  |  |
| 36 | Solid/Cribriform Carci. | 3 | D. S. | No | No | T3 | 3 - 5 | Marked | Single |  | Positive | Positive |  |
| 37 * | Cribriform Carcinoma | 14 | Siamese | Yes | No | T2 |  | Marked | Multiple | NA | Positive | Positive | DOD |
| 38 | Cribriform Carcinoma | 8 | D. S. | No |  | T2 | 5 |  |  |  |  | Positive |  |
|  | **METASTASTATIC Lesions** | | | |  |  |  |  |  |  |  |  |  |
| 39 | Lymph node |  |  |  |  |  |  |  |  |  |  |  |  |
| 40 | Lymph node (#) |  |  |  |  |  |  |  |  |  |  |  | DOD |
| 41 | Pulmonary (#) |  |  |  |  |  |  |  |  |  |  |  | DOD |

**Supplementary Table 2. Normal samples and cat mammary lesions samples haplotype evaluation. The 22 cat mammary lesions samples (CML) are group by lesion type: Benign, Malignant and Metastatic Lesions. The cat lesion samples where it was possible to analyze the respective normal tissue (*i.e.*, same individual) are indicate (Normal match CML identification). For each samples are diploid genotype are identified by it constitute haplotypes: h1 = CTACG; h2 = CTGCG; h3 = CTGCA; h4 = CTGTG; h5 = ATGCG;
h6 = CGGCG.**

| **NORMAL SAMPLES** | | **CAT MAMMARY LESION SAMPLES** | |
| --- | --- | --- | --- |
| **Normal match CML** | **Diploid genotype** | **Benign Lesions: Hyperplasic** | **Diploid genotype** |
| N1 (CML1) | h1/h1 | 1 | h1/h1 |
| N3 (CML2) | h1/h2 | 2 | h1/h2 |
|  |  | **Benign Lesions: Neoplastic** |  |
| N2 (CML6) | h1/h2 | 6 | h1/h2 |
| N4 (CML7) | h2/h2 | 7 | h2/h2 |
|  |  | 8 | h1/h5 |
|  |  | **Primary Malignant** |  |
| N5 (CML11) | h1/h3 | 9 | h1/h3 |
| N6 (CML15) | h1/h1 | 10 | h1/h1 |
| N7 (CML16) | h1/h1 | 11 | h1/h3 |
| N8 (CML17) | h1/h2 | 13 | h1/h1 |
| N9 (CML23) | h1/h1 | 14 | h1/h2 |
| N10 (CML37) | h1/h2 | 15 | h1/h1 |
|  |  | 16 | h1/h1 |
|  |  | 17 | h1/h2 |
|  |  | 19 | h1/h6 |
|  |  | 22 | h1/h2 |
| **Normal Samples** |  | 23 | h1/h1 |
| (No match CML) |  | 24 | h2/h3 |
| N11 | h1/h1 | 25 | h1/h4 |
| N12 | h1/h1 | 28 | h1/h2 |
| N12 | h1/h1 | 37 | h1/h2 |
| N14 | h2/h3 | **Metastatic Lesions** |  |
| N15 | h1/h4 | 39 (LN) | h1/h1 |
| N16 | h1/h2 | 40 (LN) | h1/h1 |

**Supplementary Table 3.** Human Splicing Finder bioinformatic tool to the prediction of alternative splice based on the sequence variants detected. (+) New site created by allelic variation; (−) Motif abolished by allelic variation; When multiple splicing enhancers/silencers sites were predicted, the number is indicated (xN means that N adjacent sites were modified). (nt) nucleotide; (ESE) Exonic Splicing Enhancers; (ESS) Exonic Splicing Silencer; (ESR) Exonic splicing regulatory sequences; (HSF) Human splicing finder; (IIE) Intron identity elements; (EIE) Exon identity elements; (CV) Consensus Values; (ΔCV) Variations of the Consensus Values; (PESE) Exonic Splicing Enhancers octamers.

| **SVs** | **SVs Genomic**  **position** | **New Potential Splice Sites** | | | **Branch Points Motif** | **Splicing Enhancers** | | **Splicing Silencers** | |
| --- | --- | --- | --- | --- | --- | --- | --- | --- | --- |
|  |  | **HSF Splicing Motif** | **HSF Variant/Reference CV (ΔCV %)** | **MaxEnt Matrice Splicing Motif (ΔCV %)** |  | **Exonic Enhancer** | **Other splicing elements** | **Exonic Silencer** | **Intronic**  **identity Elements** |
| g.153C>A | Intron 19:  Exon 19 + 63 nt |  |  |  |  | − ESE ×2  (SC35:150–157 nt) (SF2/ASF: 153–159 nt) |  |  | + IIE x4  (150/1/2/3–155/6/7/8 nt) |
| g.156T>G | Intron 19:  Exon 19 + 66 nt |  |  | 154–176 nt  (…)cactgAGG  (−72.92%) | No change | − ESE  (SC35:150–157 nt)  + ESE  (SC35:151–158 nt) | – ESR  (154--159 nt) | + Sironi ×2  (152–159 nt)  (153–160 nt) | − IIE  (154–158 nt) |
| g.224A>G | Intron 19:  Exon 19 + 134 nt  Exon 20 − 142 nt | 221–229 nt  5′ donor CCCgtgggc | 72.56/45.73  (+58.68%) | 208–230 nt  (…)gtggGCC  (−16.67%) | 219–225 nt  Site Broken  ctcccAt | + ESE ×2  (SRp40: 218–224 nt);  (SF2/ASF: 222–228 nt)  − ESE  (SF2/ASF: 221–227 nt)  + PESE  (217–224 nt) | – ESR  (221–226 nt) | − ESS  (222–229 nt)  – Sironi  (222–229 nt) | + IIE ×2  (223–228 nt)  (224–229 nt) |
| g.360C>T | Intron 19:  Exon 20 − 6 nt |  |  |  | 357–363 nt  New Site  gcc**c**cCc | + ESE  (SRp55: 356–361 nt)  − ESE  (SF2/ASF: 358–364 nt) |  | − Sironi ×3  (355–362 nt)  (356–363 nt)  (357–364 nt) | − IIE ×5  (356/7/8/9–361/2/3/4 nt)  (360–365 nt) |
| g.371G>A | Exon 20  Exon 20 + 5 nt | 368–376  5’ donor  AGCatatgt | 47.84/74.37  (−36.08%) | 368–376  AGCatatgt  (−133.5) | No change | + ESE  (hexamers:370–375 nt)  + ESE  (hexamers:366–371 nt) | + ESR  (366–371 nt) | − ESS  (365–372 nt)  − hnRNP  (367–372 nt)  − Sironi  (365–372 nt) |  |

**Supplementary Table 4.** Correlation test between cat mammary lesions clinicopathological classifiers. (C.V) Person’s coefficient values (*C* Value). (−) negative linear correlation. Person χ^2^ test *p*-values: Trends to significant(*); Significant at 0.05 level(**); Significant at 0.01 level (***).

| **Pearson Correlation** | | **Age**  **(years-old)** | **Ovary-hysterectomy** | **Hormonal treatment** | **Vascular infiltration** | **Necrosis** | **Lesion size** | **Number mitosis / field** | **Nuclear / Cellular pleomorphism** | **Number of Masses / lesion** | **Lymph node invasion** | **Clinical Outcome** |
| --- | --- | --- | --- | --- | --- | --- | --- | --- | --- | --- | --- | --- |
| Pathol. Grade | C.Value | 0.27 | −0.41 | −0.30 | 0.34 | 0.664 | 0.14 | 0.37 | 0.800 | −0.28 | 0.27 | 0.890 |
|  | *p*-values | 0.14 | 0.06 * | 0.23 | 0.24 | 0.00 *** | 0.49 | 0.10 | 0.00 ** | 0.12 | 0.38 | 0.00 *** |
| Age (years-old) | | C.Value | 0.02 | −0.08 | 0.18 | 0.05 | 0.04 | 0.34 | 0.29 | 0.22 | 0.39 | 0.32 |
|  |  | *p*-values | 0.92 | 0.76 | 0.56 | 0.84 | 0.84 | 0.15 | 0.16 | 0.26 | 0.24 | 0.40 |
| Ovary-hysterectomy | | | C.Value | 0.549 | −0.47 | −0.588 | 0.06 | −0.23 | −0.564 | 0.596 | 0.17 | −0.35 |
|  |  |  | *p*-values | 0.02 ** | 0.10 | 0.01 *** | 0.81 | 0.37 | 0.01 *** | 0.01 *** | 0.72 | 0.39 |
| Hormonal treatment | | | | C.Value | −0.57 | −0.592 | −0.17 | −0.22 | −0.36 | 0.13 | 0.17 | −0.707 |
|  |  |  |  | *p*-values | 0.08 | 0.03 ** | 0.56 | 0.49 | 0.17 | 0.64 | 0.72 | 0.05 ** |
| Vascular infiltration | | | | | C.Value | 0.606 | 0.32 | 0.39 | 0.600 | −0.36 | 1.000 | 1.000 |
|  |  |  |  |  | *p*-values | 0.05 ** | 0.28 | 0.35 | 0.04 ** | 0.20 | 0.00 *** | 0.00 *** |
| Necrosis | | | | | | C.Value | 0.48 | 0.609 | 0.837 | −0.46 | 0.38 | 0.824 |
|  |  |  |  |  |  | *p*-values | 0.06 | 0.02 ** | 0.00 *** | 0.06 | 0.36 | 0.01 *** |
| Lesion size | | | | | | | C.Value | 0.00 | 0.17 | −0.03 | 0.12 | −0.13 |
|  |  |  |  |  |  |  | *p*-values | 0.99 | 0.44 | 0.88 | 0.73 | 0.75 |
| Number mitosis / field | | | | | | | | C.Value | 0.41 | 0.14 | 0.35 | 0.83 |
|  |  |  |  |  |  |  |  | *p*-values | 0.09 | 0.58 | 0.35 | 0.09 |
| Nuclear / Cellular pleomorphism | | | | | | | | | C.Value | −0.488 | 0.26 | 0.774 |
|  |  |  |  |  |  |  |  |  | *p*-values | 0.02 ** | 0.46 | 0.02 ** |
| Number of masses / lesion | | | | | | | | | | C.Value | −0.12 | −0.25 |
|  |  |  |  |  |  |  |  |  |  | *p*-values | 0.71 | 0.46 |
| Lymph node invasion | | | | | | | | | | | C.Value | 0.58 |
|  |  |  |  |  |  |  |  |  |  |  | *p*-values | 0.42 |

**Supplementary Table 5.** Variants alleles frequency association tests between groups and subgroups of samples. The chi-square test (χ^2^ Tests) was used to test possible association allelic variation between groups or subgroup of samples with significant *p*-value < 0.05. (#) Fisher's exact test correct the association test in the cases were at least one expected cell count is less than 5 (significant *p*-value < 0.05). Pearson correlation test indicate the orientation of the association at significant *p*-value < 0.05. (BenL) Benign Lesions. (MaL) Malignant Lesions. (Hyp.BeL) Hyperplasic Benign Lesion. (Neo.BeL) Neoplastic Benign Lesion. (Pri.MaL) Primary Malignant Lesions. (Met.) Metastasis. (−) Negative correlation.

| **Sequence Variant (Genotypes)** | **Group and Subgroup of Samples**  **(group1 count *versus* group 2 count)** | **Number of Variant Allele Carriers** | **Pearson χ^2^ Tests**  **(*p*-value)** | **Pearson Correlation**  **Test (*p*-value)** |
| --- | --- | --- | --- | --- |
| 153C>A | Normal × Lesion (16 / 22) | 0 / 1 | 0.387 | 0.401 |
|  | BenL × MaL (5 / 17) | 1 / 0 | 0.059 * (>0.05 #) | 0.063 * (−) |
|  | Hyp.BeL × Neo.BeL × Pri.MaL × Met. (2 / 3 / 15 / 2) | 0 / 1 / 0 / 0 | 0.084 * (>0.05 #) | 0.381 |
|  | Neo.BeL × Pri.MaL (3 / 15) | 1 / 0 | 0.021 ** (>0.05 #) | 0.002 *** (−) |
| 156T>G | Normal × Lesion (16 / 22) | 0 / 1 | 0.387 | 0.401 |
|  | BenL × MaL (5 / 17) | 0 / 1 | 0.579 | 0.600 |
|  | Hyp.BeL × Neo.BeL × Pri.MaL × Met. (2 / 3 / 15 / 2) | 0 / 1 / 1 / 0 | 0.921 | 0.745 |
|  | Neo.BeL × Pri.MaL (2 / 15) | 0 / 1 | 0.707 | 0.728 |
| 224A>G | Normal × Lesion (16 / 22) | 9 / 14 | 0.739 | 0.924 |
|  | BenL × MaL (5 / 17) | 4 / 10 | 0.507 | 0.115 |
|  | Hyp.BeL × Neo.BeL × Pri.MaL × Met. (2 / 3 / 15 / 2) | 1 / 3 / 10 / 0 | 0.310 | 0.093 |
|  | Neo.BeL × Pri.MaL (3 / 15) | 2 / 10 | 0.270 | 0.111 |
| 360C>T | Normal × Lesion (16 / 22) | 1 / 1 | 0.816 | 0.820 |
|  | BenL × MaL (5 / 17) | 0 / 1 | 0.578 | 0.599 |
|  | Hyp.BeL × Neo.BeL × Pri.MaL × Met. (2 / 3 / 15 / 2) | 0 / 0 / 1 / 0 | 0.921 | 0.744 |
|  | Neo.BeL × Pri.MaL (3 / 15) | 0 / 1 | 0.645 | 0.727 |
| 371G>A | Normal × Lesion (16 / 22) | 4 / 5 | 0.870 | 0.875 |
|  | BenL × MaL (5 / 17) | 2 / 3 | 0.294 | 0.317 |
|  | Hyp.BeL × Neo.BeL × Pri.MaL × Met. (2 / 3 / 15 / 2) | 1 / 1 / 3 / 0 | 0.209 | 0.106 |
|  | Neo.BeL × Pri.MaL (3 / 15) | 1 / 3 | 0.612 | 0.517 |

© 2012 by the authors; license MDPI, Basel, Switzerland. This article is an open access article distributed under the terms and conditions of the Creative Commons Attribution license (http://creativecommons.org/licenses/by/3.0/).
